# Colorectal Cancer Screening Barriers in Persistent Poverty Communities: A Qualitative Analysis

**DOI:** 10.1093/geroni/igaf122.3563

**Published:** 2025-12-31

**Authors:** Margaret Salisu

**Affiliations:** SUNY @ Downstate, Bayshore, New York, United States

## Abstract

**Background:**

Colorectal cancer (CRC) is the second leading cause of cancer death in the U.S., yet screening uptake is low in communities experiencing persistent poverty. Black and Hispanic adults face disproportionate burden due to intersecting socioeconomic and structural inequities. Guided by the Andersen Behavioral Model, this study explored multilevel barriers and facilitators to CRC screening.

**Methods:**

Nineteen semi-structured interviews and two focus groups (n = 25) were conducted with Black and Hispanic adults aged 45–70, along with provider key informant interviews. Data were analyzed thematically using Andersen’s domains of predisposing, enabling, and need factors.

**Results:**

Predisposing factors included limited awareness, cultural stigma, fear of diagnosis, and mistrust of the healthcare system. During interviews, several participants reported wanting to get screened after learning more, demonstrating the potential of real-time education. Enabling barriers included cost concerns, transportation challenges, scheduling complexity, and inadequate accommodations for people with disabilities. Need factors reflected divergent perceptions of risk: individuals with family history or symptoms expressed urgency, while others delayed screening due to low perceived vulnerability or fatalistic beliefs. Providers emphasized systemic inefficiencies, while patients highlighted emotional and cognitive barriers.

**Conclusions/Implications:**

CRC screening disparities in persistent poverty communities are driven by informational, structural, and psychosocial barriers. Findings highlight the need for culturally tailored, disability-inclusive interventions that build trust, simplify access, and leverage community-based education. Brief, personalized conversations may serve as powerful catalysts for screening uptake. These insights inform equity-focused strategies to reduce CRC disparities in underserved aging populations.

